# Age, Arterial Stiffness, and Components of Blood Pressure in Chinese Adults

**DOI:** 10.1097/MD.0000000000000262

**Published:** 2014-12-02

**Authors:** Meili Zheng, Xiping Xu, Xiaobin Wang, Yong Huo, Xin Xu, Xianhui Qin, Genfu Tang, Houxun Xing, Fangfang Fan, Wei Cui, Xinchun Yang

**Affiliations:** From the Heart Center, Beijing Chao-Yang Hospital, Capital Medical University, Beijing, China (MZ, XY); Guangdong Institute of Nephrology, Southern Medical University, Guangzhou, China (XX, XX, XQ); Center on the Early Life Origins of Disease, Department of Population, Family and Reproductive Health, Johns Hopkins, University Bloomberg School of Public Health, Baltimore, MD, USA (XW); Cardiology Department, Peking University First Hospital, Beijing, China (YH, FF); and Institute of Biomedicine, Anhui Medical University, Hefei, China (GT, HX, WC).

## Abstract

Blood pressure (BP) changes with age. We conducted a cross-sectional study in rural Chinese adults to investigate: (1) what is the relationship between age, arterial stiffness, and BP in Chinese men and women; and (2) to what degree can the age–BP relationship be explained by arterial stiffness, controlling for other covariables.

These analyses included a total of 1688 subjects (males/females: 623/1065), aged 40 to 88 years. Among them, 353 (20.9%) had hypertension (defined as systolic blood pressure (SBP) ≥140 mm Hg or diastolic blood pressure (DBP) ≥90 mm Hg). Arterial stiffness was measured by brachial–ankle pulse wave velocity (baPWV).

baPWV appeared to be more strongly correlated with BP (including SBP, DBP, mean arterial pressure [MAP], pulse pressure [PP]) than age (*P* < 0.001 for comparisons between Spearman correlation coefficients). Furthermore, baPWV was associated with BP (including SBP, DBP, MAP, and PP) and risk of hypertension in a dose–response fashion, independent of age; in contrast, the age–BP associations were either attenuated or became negative after adjusting for baPWV.

Arterial stiffness appears to be an independent contributor to hypertension, even after adjusting for age and other covariables. In contrast, age–BP associations became attenuated or negative after adjusting for baPWV. The utility of baPWV as a diagnostic, prognostic, and therapeutic indicator for hypertension warrants further investigation.

## Abstract 2

Supplemental Digital Content is available in the text

## INTRODUCTION

Essential hypertension is the most common chronic disease in the Chinese population, affecting 260 million people in China. Hypertension can lead to stroke, coronary heart disease, and other vascular complications, which are the leading causes of morbidity and mortality in China.^1^

A number of studies^2–4^ have shown an increase in the prevalence of hypertension with advancing age and the predominance of systolic hypertension in the elderly^5,6^ (a greater increase in systolic blood pressure (SBP) than diastolic blood pressure (DBP) during the middle adult years, whereas while SBP continues to rise until the eighth or ninth decade, DBP tends to remain constant or decline after the fifth or sixth decade^7^). One likely explanation for the differential pattern of SBP versus DBP with aging is a progressive “stiffening” of the arteries. Indeed, recent studies have demonstrated that arterial stiffness precedes hypertension and promotes a progressive increase in SBP.^8–12^ One study^13^ suggested (in 2 figures) that SBP actually decreased with age at the same arterial stiffness level (measured by brachial–ankle pulse wave velocity [baPWV]) after age 40 years.

Our study is the first to unravel the inter-relationships between age, arterial stiffness and components of BP in a Chinese population. In this study, arterial stiffness was assessed by baPWV,^11,12^ which correlated well with carotid–femoral pulse wave velocity (cfPWV)^8,10^ and other indexes.^14^ We aimed to investigate: (1) what is the relationship between age and blood pressure (BP) and between arterial stiffness and BP in rural Chinese men and women; and (2) to what degree can the age–BP relationship be explained by arterial stiffness, controlling for other covariables.

## METHODS

### Study Population

In the present study, we included rural subjects from Lianyungang of Jiangsu province and Anqing of Anhui province. Exclusion criteria included a reported history of myocardial infarction, stroke, heart failure, cancer, and/or serious mental disorder; and an unwillingness to participate or a difficulty completing the survey. All patients gave written informed consent. Study complied with the Helsinki Declaration and was approved by the Ethics Committee of the Institute of Biomedicine, Anhui Medical University, Hefei, China.

**FIGURE 1:**
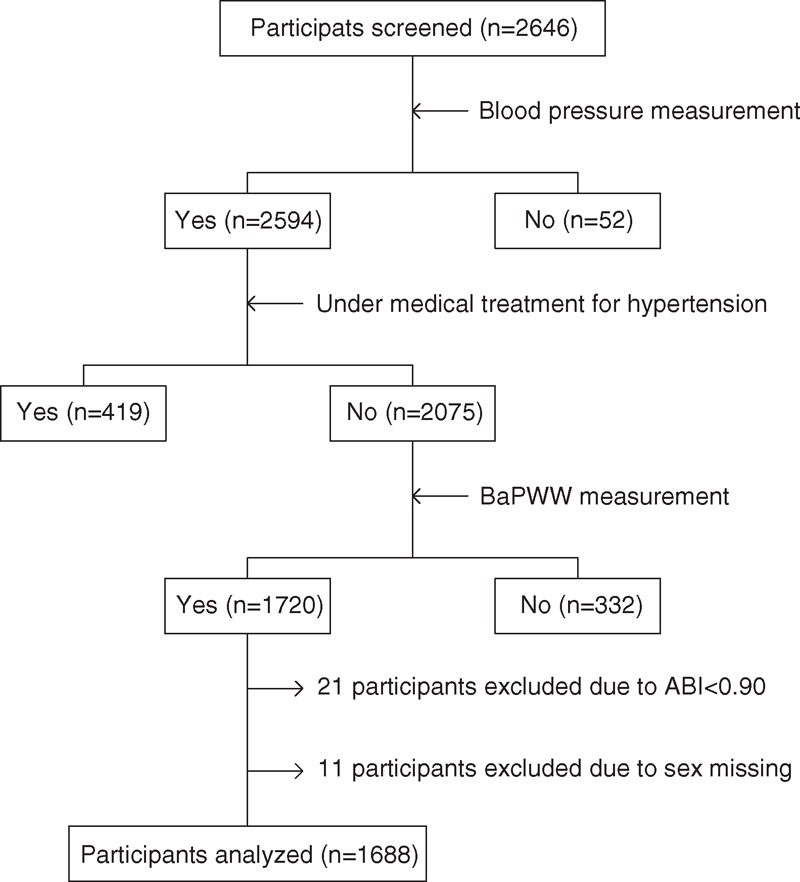
Flow diagram of screening and enrollment of participants. BaPWV = brachial–ankle pulse wave velocity, ABI = ankle/brachial systolic blood pressure index.

A total of 2646 subjects were screened in this study. Among them, 2075 participants completed BP measurement and were without anti-hypertensive treatment. Of those, 1720 subjects underwent baPWV measurement and 21 individuals with ankle/brachial systolic BP indexes <0.90 as well as 11 individuals with sex missing were excluded (the flowchart is provided as Figure 1).

### Blood Pressure Measurements

Participants were placed in the sitting position with their right arms supported at the level of the heart. Automatic digital sphygmomanometers (Omron HEM 705IT device; Omron Health Care) were used to measure the BP of the participants by trained volunteers recruited from local medical colleges. Three minutes of rest was given to the participant in between 3 successive BP readings. We used the average of the 3 readings as the final BP value. Hypertension was defined as a BP of 140 mm Hg systolic or 90 mm Hg diastolic or higher.

### Brachial–Ankle Pulse Wave Velocity Measurements

Brachial–ankle pulse wave velocity (baPWV) was automatically measured with form PWV/ABI instruments (form PWV/ABI, BP-203RPE; Omron-Colin, Japan) by trained volunteers recruited from local medical colleges. Occlusion and monitoring cuffs matched with oscillometric sensors were wrapped around subjects’ upper arms and ankles, and pulse volume waveforms of the bilateral brachial and tibial arteries were recorded simultaneously to determine the time interval between the initial increase in brachial and tibial waveforms (transit time, ΔTba). The transmission distance from the brachium to the ankle was calculated according to body height. The path length from the suprasternal notch to the brachium (Lb) was obtained using the following equation: Lb = 0.2195 × height of the patient (in cm) −2.0734. The path length from the suprasternal notch to the ankle (La) was obtained using the following equation: La = (0.8129 × height of the patient [in cm] + 12.328). The baPWV value was calculated as the ratio of transmission distance from the brachium to the ankle divided by the transit time: baPWV = (La − Lb)/ΔTba. After obtaining bilateral baPWV, the higher one was used for analysis. Ankle brachial index (ABI), which is the ratio of SBP in the ankle to that in the brachial artery, was evaluated simultaneously.

### Questionnaire

All participants were administered a standardized questionnaire that provided information related to occupation, medical history, past and current medications, and personal habits such as cigarette and alcohol consumption.

### Laboratory Tests

After 12 to 15 hours of fasting, a venous blood sample was obtained from each subject. Serum or plasma samples were separated within 30 minutes of collection and were stored at −70 °C. All collected blood samples were used to measure glucose, homocysteine, creatinine, and lipids (including total cholesterol [TC], high-density lipoprotein cholesterol, and triglycerides).

### Statistical Analysis

Data are expressed as percentages or mean ± SD. The differences between groups were checked by χ^2^ test for categorical variables or by independent *t*-test for continuous variables. Since the baPWV was non-normally distributed, we re-set all the values >2500 cm/second (n = 33) to 2500 cm/second to minimize the undue influence of extremely high values for baPWV. Spearman correlation was used to assess univariate associations. After grouping subjects into age categories (C1, <60 years; C2, 60–69 years; C3, ≥70 years) or baPWV quartiles (Q1, <1302 cm/s; Q2, 1302–1458 cm/s; Q3, 1459–1681 cm/s; Q3, ≥1682 cm/s), we used linear and logistic regression to investigate the associations between age or baPWV with BP (including SBP, DBP, MAP, and PP) and risk of hypertension. Trend tests were computed by modeling the baPWV quartiles or age category medians as continuous variables. All analyses were performed using EmpowerStats (http://www.empowerstats.com) and the statistical package R.

## RESULTS

### Subject Characteristics

The present study included 1688 subjects (age: 62.2 ± 7.3 years, range 40–88 years, male/female 623/1065). Characteristics of the study population are listed in Table 1. A total of 353 (20.9%) subjects had hypertension, and, compared with normotensives, hypertensives had higher SBP, DBP, mean arterial pressure (MAP), pulse pressure (PP), baPWV, heart rate (HR), body mass index (BMI), TC, triglycerides, and homocysteine. There were no significant differences in gender distribution, glucose, high-density lipoprotein cholesterol (HDL-C), creatinine, or smoking and alcohol consumption.

**TABLE 1:**
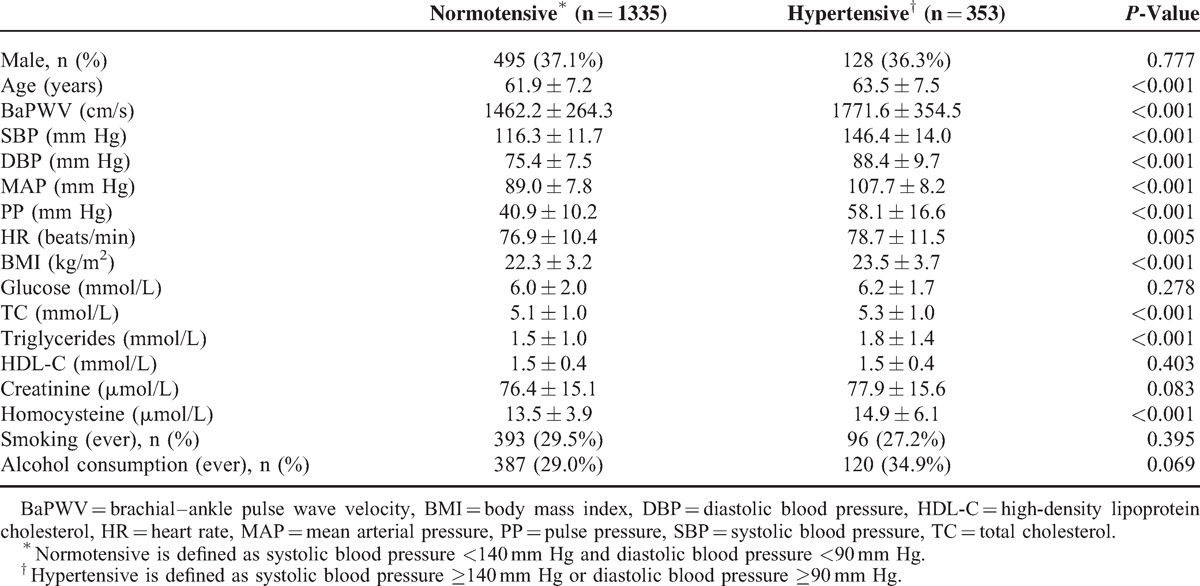
Characteristics of the Study Participants

### Univariate Correlations Between Blood Pressure, Age, and BaPWV

Both age and baPWV were significantly correlated with SBP and DBP; correlation coefficients were 0.54 for baPWV and SBP, 0.28 for baPWV and DBP, 0.18 for age and SBP, and −0.08 for age and DBP (*P* all <0.001 for Spearman correlation coefficients) (see Table, Supplemental Digital Content 1 http://links.lww.com/MD/A100, which shows Spearman correlations between BP, age, and baPWV). BaPWV was more strongly correlated with SBP and DBP than age (*P* all <0.001), which is consistent with a previous study^15^; correlation coefficients were 0.65 for baPWV and SBP, 0.66 for baPWV and DBP, 0.27 for age and SBP, and 0.26 for age and DBP (*P* all <0.001). BaPWV appeared to be a better predictor of SBP and DBP than age. BaPWV also had a stronger correlation with SBP than DBP in the present study (*P* < 0.001). The correlation coefficient for age and baPWV was around 0.50 in ours as well as in previous studies.^15,16^ The univariate correlations separately performed in normotensive and hypertensive patients were shown in Supplemental Digital Content 2 (http://links.lww.com/MD/A100).

### Dose-Dependent Increase of Blood Pressure With BaPWV

In univariate models, we observed a significant and progressive increase in BP (including SBP, DBP, MAP, and PP) with baPWV quartiles (*P* for trend all <0.001), suggesting a dose-dependent increase of BP with baPWV. Furthermore, the effect of baPWV on SBP was much stronger than that on DBP. These associations remained unchanged after further adjustment for age, HR, BMI, glucose, TC, triglycerides, high-density lipoprotein cholesterol, homocysteine, creatinine, and smoking and alcohol consumption (Table 2).

**TABLE 2:**
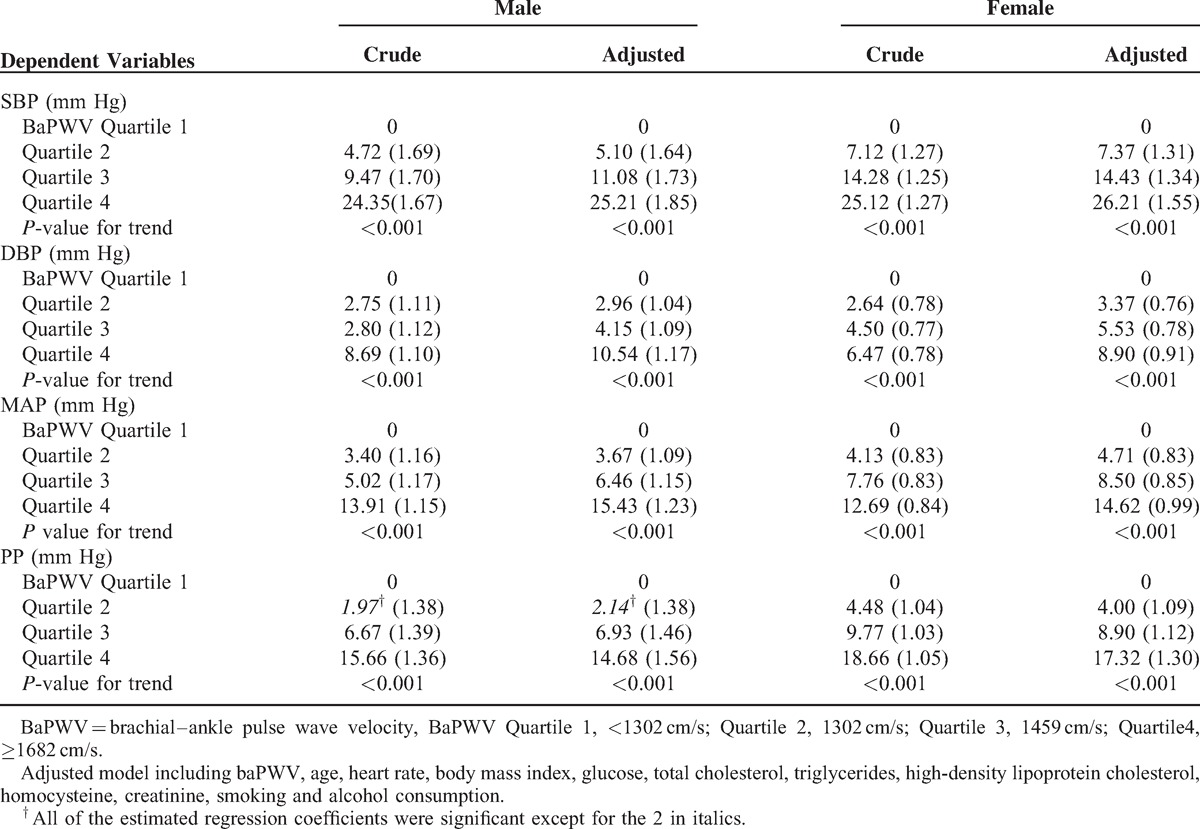
Linear Regression Analysis of BaPWV in Relation to Components of Blood Pressure

### Different Effects of Age on Components of Blood Pressure

SBP increased with age in univariate analysis whereas baPWV-adjusted estimated regression coefficients for SBP across age categories turned out to be negative in both genders, and further adjustments for other covariables (including HR, BMI, glucose, TC, triglycerides, high-density lipoprotein cholesterol, homocysteine, creatinine, smoking, and alcohol consumption) attenuated but did not reverse the negative effects. DBP decreased with age consistently before and after adjustments; however, further adjustments for baPWV and other covariables enhanced the negative effects, which appear to be much stronger than the effect on SBP. In multivariate models, MAP significantly and progressively decreased with age after adjustment for baPWV in both genders (*P* for trend <0.001), and further adjustments for other covariables only mildly attenuated the negative effects. The increase in PP with advancing age was clearly attenuated after adjustment for baPWV, and was barely unchanged after further adjustment for other covariables (Table 3). Additionally, the *P* values for trend for SBP (in male) and PP (in female) lose statistical significance after adjustment for BaPWV + other covariables but not after adjustment for BaPWV alone.

**TABLE 3:**
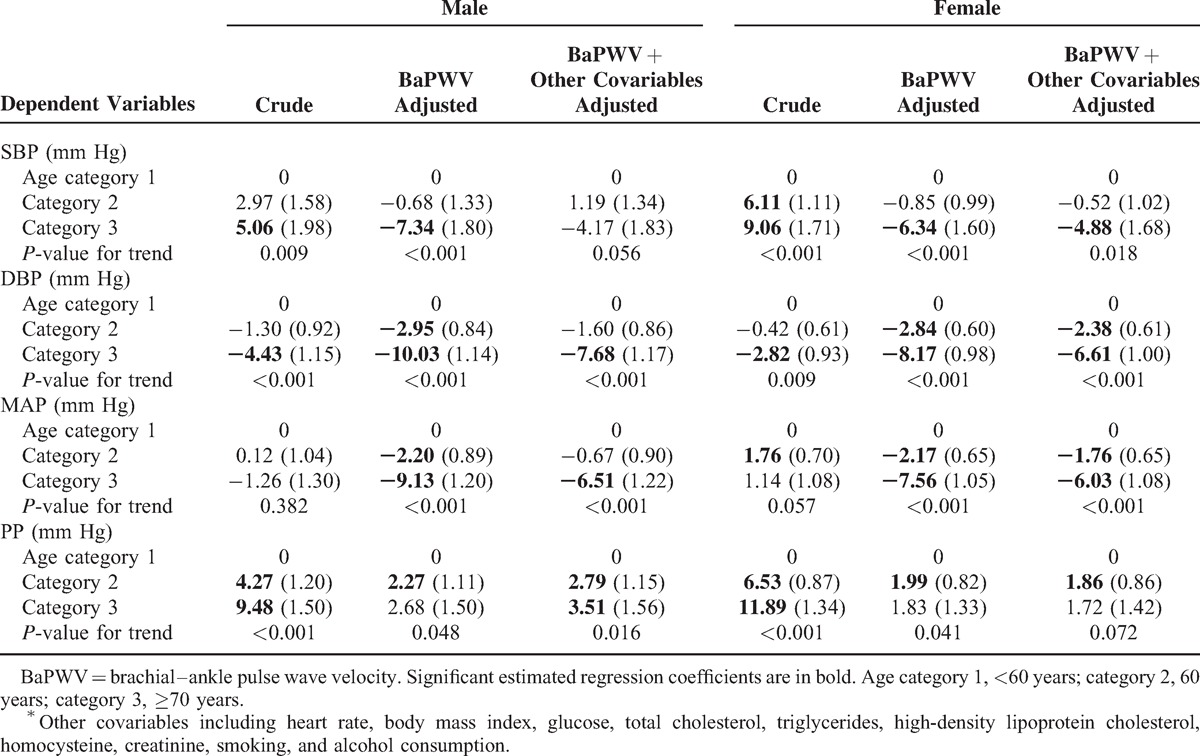
Linear Regression Analysis of Age in Relation to Components of Blood Pressure

### BaPWV is a Stronger Contributor to Hypertension Than Age

In multivariate models including age, baPWV, HR, BMI, glucose, TC, triglycerides, high-density lipoprotein cholesterol, homocysteine, creatinine, and smoking and alcohol consumption, the odds ratios for hypertension increased across baPWV quartiles, reaching 44.8 (95% confidence interval [CI], 14.1–142.0) in males and 21.7 (95% CI, 11.0–43.0) in females for the top quartiles as compared to the bottom quartiles (*P* for trend all <0.001) (Table 4). Whereas the odds ratios for hypertension tended to decrease across age categories, reaching 0.42 (95% CI, 0.19–0.92) in males for the top quartiles as compared with the bottom quartiles (*P* for trend = 0.033); and 0.56 (95% CI, 0.29–1.05) in females (*P* for trend = 0.117). As such, baPWV appears to be a stronger predictor for hypertension than age.

**TABLE 4:**
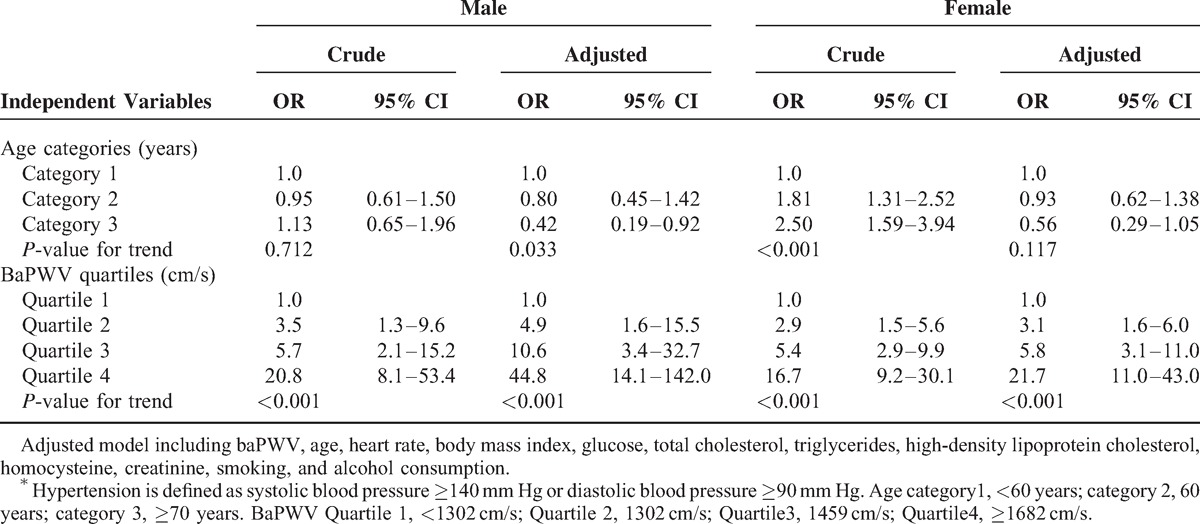
Logistic Regression Analysis of Age and BaPWV in relation to the Risk of Hypertension^∗^

## DISCUSSION

In this cross-sectional study of 1688 relatively healthy subjects, we found baPWV to be positively associated with BP (including SBP, DBP, MAP, and PP) and risk of hypertension in a dose–response fashion, independent of age. In contrast, age tended to be negatively associated with BP (including SBP, DBP, and MAP) and risk of hypertension after adjusting for baPWV. Our findings suggest that arterial stiffness appears to be an independent contributor to hypertension nor a progressive increase in BP, and that other age-related effects concealed by the effect of arterial stiffness, tended to decrease BP in the elderly.

The Framingham Heart Study^7^ identified a linear rise in SBP from age 30 through 84 years and concurrent increases in DBP and MAP; whereas after age 50 to 60 years, DBP declined, PP rose steeply, and MAP reached an asymptote. To explain the late age-related decline in DBP, a hypothesis, namely an age-related decrease in cardiac output was denied because it is inconsistent with a concurrent late rise in SBP. However, it is supported by our results, which show late declines in SBP and DBP with aging. Our results are also consistent with a previous study^13^ that found associations between age, baPWV, and SBP with results suggesting that SBP decreases with age at the same baPWV level after age 40 years. Interestingly, the Framingham Heart Study also considered calculated MAP underestimating of vascular resistance after age 50 years. This conclusion was based on results showing a leveling off of MAP after age 50 to 60 years in the Framingham Heart Study, which was in conflict with evidence that vascular resistance continues to rise with age.^17,18^ Actually, the key point neglected here is that MAP not only increases as age-related vascular resistance increases but also decreases with age-related cardiac output declines. We found a dose-dependent decrease in MAP with aging after adjusting for baPWV.

Age-related changes in BP are determined by both age-related increases in arterial stiffness^17,18^ and age-related decreases in cardiac output,^19^ and arterial stiffening appears to increase SBP much more than DBP (speculated from our results showing relationships between baPWV and BP) while cardiac output declines appear to decrease SBP much less than DBP (speculated from our results showing relationships between age and BP after adjustment for baPWV). This may explain why there are so many healthy elderly normotensives: age-related increases in BP due to arterial stiffening are offset by age-related decreases in BP due to a decline in cardiac output. Furthermore, an increase in arterial stiffness is an independent contributor to an increase in BP. We found a dose-dependent increase in BP with arterial stiffness independent of age. This may also explain why so many young hypertensives (not secondary) exist—severe arterial stiffness alone may be enough to promote a progressive increase in BP.

Previous studies have demonstrated that arterial stiffness (either cfPWV or baPWV) predicts incident hypertension^11,12^ and is an independent determinant of longitudinal increases in SBP.^10,13^ In fact arterial stiffness not only predicts hypertension but also the risk of cardiovascular events or even total mortality. A meta-analysis^20^ showed that baPWV may predict the risk of cardiovascular events and mortality, and those with higher baPWV had an increased risk of cardiovascular events (RR = 2.89, 95% CI 1.99–4.20), cardiovascular mortality (RR = 7.68, 95% CI 3.91–15.07) and all-cause mortality (RR = 2.48, 95% CI 1.82–3.37). Another meta-analysis showed the pooled RRs of total CV events, CV mortality, and all-cause mortality to be 2.26 (95% CI: 1.89–2.70, 14 studies), 2.02 (95% CI: 1.68–2.42, 10 studies), and 1.90 (95% CI: 1.61–2.24, 11 studies), respectively, for subjects with high versus low cfPWV.^21^ Consequently, anti-stiffness interventions will not only decrease high BP, but also improve the prognosis of individuals with cardiovascular diseases. Finally, arterial stiffness may serve as a useful diagnostic and therapeutic indicator of hypertension or other cardiovascular diseases.

The use of baPWV has been criticized since the pulse wave does not travel directly from the brachial arteries to the post-tibial arteries in the same arterial tree. However, the same argument can be made for the well-established cfPWV. CfPWV measures the velocity of the pulse wave from carotid to femoral arteries, and these 2 arteries are not directly connected in the same arterial tree. Another issue pertaining to cfPWV is that there has been no consensus in terms of how the arterial path length should be measured, and hence different investigations have employed different cfPWV measurements,^22,23^ which has led to wide variation in cfPWV values.^24,25^ Actually, baPWV correlates well with cfPWV with a correlation coefficient around 0.75 to 0.87^26–28^; however, because baPWV assesses the mechanical property of a large territory covering both the large-sized central elastic and medium-sized peripheral muscular arteries, baPWV may be more representative than cfPWV of arterial load. Previous studies have shown that, compared with cfPWV, baPWV was not only somewhat more strongly related to various risk factors for cardiovascular disease (such as SBP, DBP, MAP, and PP),^29^ but displayed stronger associations with the presence of coronary calcium^30^ and left ventricular mass.^28^ In the present study, we preferred baPWV since it offers an automated, non-invasive measurement that can be easily applied in general population studies.^31^

The main limitation of this study was its cross-sectional design, which does not allow for causal relationships to be confirmed. Replication using a prospective design is needed to determine the nature of the relationships between age, arterial stiffness, and BP.

In summary, increased arterial stiffness with age is an independent contributor to increases in BP; however, this may not represent all of the age-related effects on BP, such as age-related cardiac output decline, which may decrease BP in the elderly.

The most important clinical implications that can be derived from this study are that: (1) anti-stiffness interventions are necessary since arterial stiffness is an independent contributor to hypertension and progressive increases in BP; (2) calculated MAP may serve as a surrogate measurement for join forces of arterial stiffness and cardiac output in the elderly; and (3) a late fall in DBP may be an indicator of cardiac output decline in the elderly.
